# Prime editing in hematopoietic stem cells—From *ex vivo* to *in vivo* CRISPR-based treatment of blood disorders

**DOI:** 10.3389/fgeed.2023.1148650

**Published:** 2023-03-10

**Authors:** Jonas Holst Wolff, Jacob Giehm Mikkelsen

**Affiliations:** Department of Biomedicine, Aarhus University, Aarhus, Denmark

**Keywords:** prime editing, pegRNA, CRISPR, hematopoietic stem cells, HSPCs, primary immunodeficiencies, blood disorders

## Abstract

Prime editing of human hematopoietic stem cells has the potential to become a safe and efficient way of treating diseases of the blood directly in patients. By allowing site-targeted gene intervention without homology-directed repair donor templates and DNA double-stranded breaks, the invention of prime editing fuels the exploration of alternatives to conventional recombination-based *ex vivo* genome editing of hematopoietic stem cells. Prime editing is as close as we get today to a true genome editing drug that does not require a separate DNA donor. However, to adapt the technology to perform *in vivo* gene correction, key challenges remain to be solved, such as identifying effective prime editing guide RNAs for clinical targets as well as developing efficient vehicles to deliver prime editors to stem cells *in vivo*. In this review, we summarize the current progress in delivery of prime editors both *in vitro* and *in vivo* and discuss future challenges that need to be adressed to allow *in vivo* prime editing as a cure for blood disorders.

## Introduction

The human hematological system is host to a multitude of monogenic blood disorders with a wide range of biological and clinical manifestations, affecting all blood cell lineages depending on the gene at fault. Given that a common population of hematopoietic stem cells (HSCs) gives rise to all blood linages, stem cell transplantation has typically been an option for treatment of blood disorders, but the risk of graft-versus-host disease and general morbidity (reviewed in Alexander and Greco (2022)) combined with difficulties in finding matched donors have pushed the field towards finding ways of curing the patient’s own faulty HSCs. Over the last 30 years, the ability to isolate and manipulate autologous HSCs from patients *ex vivo* has allowed conventional retro- or lentiviral gene therapies to be developed with promising results reported in phase I/II clinical trials, although insertional mutagenesis remains a concern of these gene therapies (reviewed in Wolff and Mikkelsen (2022)). More recently, development of the CRISPR/Cas9 technology (Jinek et al., 2012; Cong et al., 2013; Jinek et al., 2013; Mali et al., 2013) has pushed the limits for genetic intervention, facilitating targeted, template-guided correction of disease-causing genetic variants and opening new paths for treatment of diseases of the blood. In 2016, pioneering work by both Dever and colleagues (Dever et al., 2016) and DeWitt and coworkers (DeWitt et al., 2016) demonstrated *ex vivo* CRISPR/Cas9-based gene correction of the *HBB* gene in human CD34^+^ hematopoietic stem and progenitor cells (HSPCs), offering a potential treatment of sickle cell disease. Since then, numerous reports have described *ex vivo* Cas9-directed gene correction as a potential treatment of various blood disorders, including X-linked chronic granulomatous disease (De Ravin et al., 2017; De Ravin et al., 2021), X-linked hyper-IgM syndrome (Kuo et al., 2018; Vavassori et al., 2021), SCID-X1 (Schiroli et al., 2017; Pavel-Dinu et al., 2019), Wiskott-Aldrich Syndrome (Rai et al., 2020), β-thalassemia (Cromer et al., 2021; Pavani et al., 2021) and sickle cell disease (SCD) (Dever et al., 2016; DeWitt et al., 2016; Hoban et al., 2016; Antony et al., 2018; Vakulskas et al., 2018; Martin et al., 2019; Park et al., 2019; Romero et al., 2019; Lattanzi et al., 2021; Wilkinson et al., 2021). Notably, in August of 2022 the first patient received a dose of the Graphite Bio-developed GPH101 genome editing therapy for sickle cell disease as part of the CEDAR phase I/II clinical trial (NCT04819841) (https://graphitebio.com). The CEDAR trial was halted, however, in January of 2023 after a severe adverse event was reported in the first patient (https://graphitebio.com).

*Ex vivo* genome editing of HSPCs is still without a doubt a versatile and effective treatment option of monogenic blood disorders. However, clinical protocols require large amounts of stem cells to be mobilized from patients using granulocyte colony-stimulating factor (G-CSF) or plerixafor, a procedure which can be life-threatening for patients (Adler et al., 2001; Grigg, 2001; Boulad et al., 2018; Lagresle-Peyrou et al., 2018). Furthermore, manipulation and expansion of HSPCs *ex vivo* is a demanding and time-consuming process, which is expensive and seems to confine the desire of the biotech industry to scrutinize and push the development of *ex vivo* genome editing therapeutics. Due to the complexity of *ex vivo* handling of stem cells in combination with carrying out gene editing to therapeutically relevant levels, attention is increasingly attracted to new genetic drug designs and delivery technologies, which could potentially facilitate *in vivo* gene correction. Among numerous compelling questions, the most crucial is probably whether gene editing tool kits can be ferried to stem cells in patients in a manner that allows safe and potent gene correction in a cell type-specific manner.

One can hardly claim that *in vivo* genome editing is an under-researched area, but researchers within the field of genome editing of blood disorders have been somewhat reluctant to move from successful *ex vivo* therapies to less established approaches for *in vivo* editing. Such hesitation is obviously rooted in the current lack of methods for safe, targeted, and potent *in vivo* delivery, but also reflects some of the intrinsic challenges of conventional CRISPR/Cas9-based genome editing, including excess by-product formation (indels, translocations, and inversions), potential chromothripsis, and extensive off-target effects, which arise as a result of the Cas9-induced DNA double-stranded break (DSB) (Cradick et al., 2013; Fu et al., 2013; Lin et al., 2014). Safety is further challenged by the toxicity observed in HSCs, which is—at least in part—caused by the p53 response triggered by the DSB (Schiroli et al., 2019; Ferrari et al., 2020). Furthermore, for Cas9-based gene correction, a donor template for homology-directed repair (HDR) is required, which is usually supplied either as ssODNs or using recombinant AAV6 (rAAV6) (DeWitt et al., 2016), further adding elements of toxicity and complexity with impact on overall efficacy (Ferrari et al., 2022).

## *In vivo* prime editing of HSCs is on the horizon

In an ideal world, a *true* genome editing *drug* would be independent of DSBs and donor templates, produce no by-products, and be devoid of off-target effects. Development of base editors (BEs) represented a major leap towards DSB-free genome editing. By fusing a catalytically dead Cas9 or a Cas9 nickase to different deaminases, it became possible to install transition mutations without generating DSBs (Komor et al., 2016; Gaudelli et al., 2017). Since, the continued evolution of BEs has increased efficacy, allowing the technology to be used in pre-clinical studies of *ex vivo* HSPC genome editing therapies for SCD and Fanconi anemia (Zeng et al., 2020; Newby et al., 2021; Siegner et al., 2022). However, while results so far seem promising, BEs are challenged by unwanted by-stander base conversions when multiple targetable cytosines or adenines are present at the target site, as well as both sgRNA-dependent and -independent off-target base conversions (Grünewald et al., 2019; Rees et al., 2019). Additionally, whereas recent engineering of BEs has allowed the installation of transversion mutations (Molla et al., 2020; Zhao et al., 2021), BEs are still not able to install small deletions or insertions. To this end, the development of the prime editing technology by Anzalone and coworkers in 2019 (Anzalone et al., 2019) provided yet another step towards DSB-free genome editing allowing precise installation of both transitions, transversions, insertions, and deletions. Prime editing relies neither on DSBs nor on donor templates, and thus prime editing is associated with very little cellular toxicity and virtually no indel formation, off-target editing, or by-stander mutations (Anzalone et al., 2019; Kim et al., 2020; Chen et al., 2021; Nelson et al., 2021). Prime editing is as close as we get today to a true genome editing drug, which may potentially, with further development, support *in vivo* genome editing to be performed with yet unprecedented precision and safety.

**FIGURE 1:**
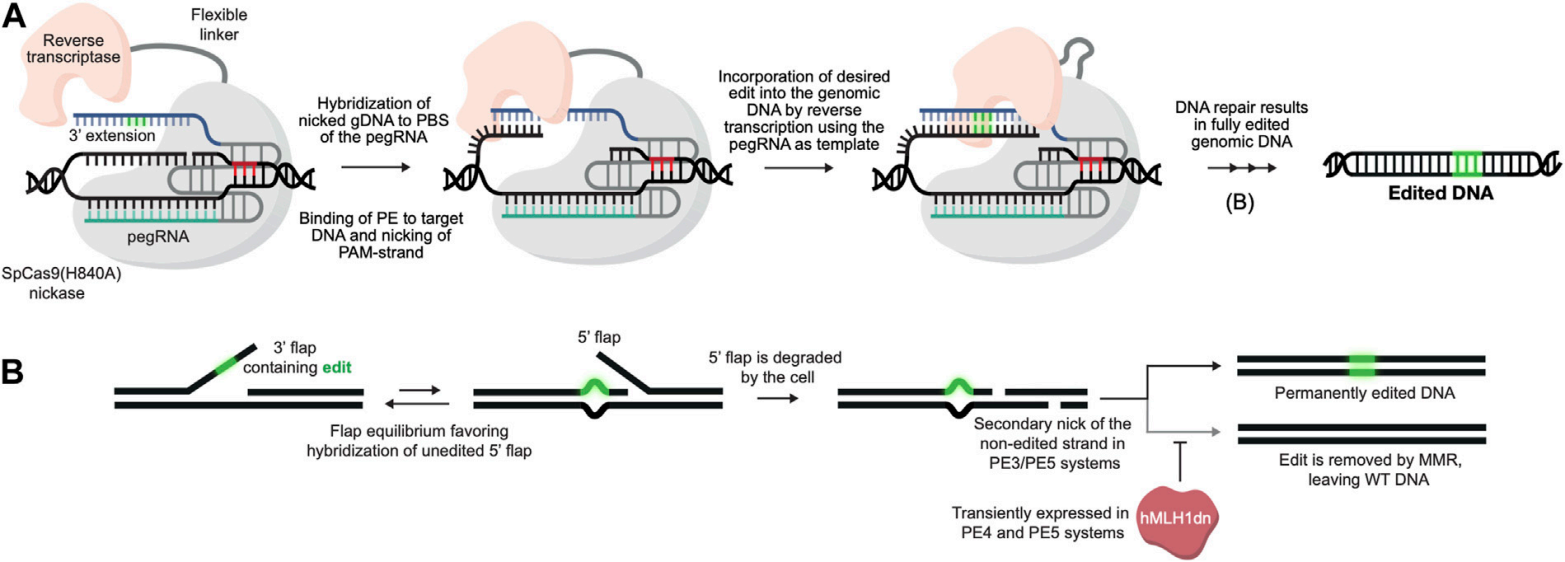
DSB-free genome editing using prime editors. **(A).** Prime editing is initiated with the binding of the PE/pegRNA complex to its genomic target DNA. The SpCas9(H840A) nickase then nicks the PAM-containing strand, following which the 3′ end of the nicked DNA strand hybridizes with the complementary primer binding site (PBS) of the pegRNA, allowing the RT to use the genomic DNA strand itself as a primer for reverse transcription. The desired edit (green) is then reverse transcribed from the reverse transcriptase template (RTT) of the pegRNA and incorporated directly into the genomic DNA strand. **(B)**. Following reverse transcription by the PE/pegRNA complex, the edited DNA strand is copied onto the non-edited strand through DNA repair by the cell. To bias repair towards the non-edited strand, the third and fifth generation of prime editors (PE3 and PE5, respectively) use an auxiliary nicking sgRNA to generate a nick in the non-edited strand. In the fourth (PE4) and fifth generation of prime editors a dominant negative hMLH1 protein is also transiently expressed to inhibit mismatch repair (MMR), further increasing editing efficacies.

Prime editing is based on the CRISPR/Cas9 system and is capable of installing virtually all small types of alterations in the genome without the need for either donor templates or DSBs (Anzalone et al., 2019). In its simplest form, the prime editing technology requires only a programmable Cas9 nickase (typically SpCas9(H840A)) (Jinek et al., 2012) fused to a reverse transcriptase (RT), which is guided by 3′ extended guide RNAs (prime editing guide RNAs or pegRNAs) (Figure 1A). What makes prime editing stand out from previous CRISPR/Cas9 technologies is that the pegRNA not only directs the Cas9 to its target site, but also acts as template for the reverse transcriptase, thereby encoding the desired edit to be written directly into the genome (Figure 1B). Since the initial report of prime editing in 2019, several improvements have been made to prime editors, including i) use of auxiliary nicking sgRNAs (ngRNAs) to nick the non-edited strand in the PE3 and PE5 systems (Anzalone et al., 2019), ii) optimization of the protein architecture (inclusion of additional NLS signals, activity-enhancing amino acid substitutions as well as codon optimization in SpCas9(H840A) and MMLV-RT) (Chen et al., 2021), iii) transient inhibition of mismatch repair in PE4 and PE5 systems (Chen et al., 2021) and iv) engineering of pegRNAs (epegRNAs) with increased stability (Nelson et al., 2021). The prime editing technology holds the promise of revolutionizing the genome editing field, and with the most recent improvements (the fifth generation of prime editors emerged at the end of 2021), the technology is getting even more powerful and reaching even higher standards for DSB-free genome editing (Chen et al., 2021; Nelson et al., 2021). Despite the potential of prime editing, reports on successful use for *ex vivo* genome editing of HSPCs have been few. Notably, data disclosed in relation to the initial public offering of Prime Medicine (https://primemedicine.com) suggest that *ex vivo* prime editing of human CD34^+^ HSPCs can be quite effective and may support treatment of certain blood disorders. Indeed, a very recent study showed that human adenoviral vector-mediated delivery of prime editors to HSCs allows potent prime editing both *ex vivo* and *in vivo*, thereby providing the first proof-of-principle that prime editing of HSCs has the potential to be used as a treatment for blood disorders (Li et al., 2023). Still, progress in the field has been relatively slow, which may reflect different aspects of the technology. One explanation is linked to complexity of the pegRNA design and the inherent difficulties identifying effective pegRNAs for new targets. Whereas sgRNAs for conventional CRISPR-Cas9-based gene editing as well as base editing only requires design of the spacer sequence, pegRNAs have several parameters affecting effective gene editing. In addition to the spacer sequence, pegRNAs also contain a 3’ extension that contains the primer binding site (PBS) as well as the reverse transcriptase template (RTT), both of which are crucial for allowing the MMLV-RT to install the desired genomic changes. Furthermore, current evidence suggests that there is little predictability in pegRNA design and efficacy, and although efforts have been made to further elucidate design criteria of pegRNAs, only little improvement has been made in this regard since the initial description of prime editors by Anzalone and colleagues (Anzalone et al., 2019; Kim H. K. et al., 2021). Thus, identifying novel effective pegRNAs is currently accomplished by labor-intensive manual screening, which has been somewhat aided by pegRNA design tools developed by us and others (Anderson et al., 2021; Li Y. et al., 2021; Chow et al., 2021; Hsu et al., 2021; Hwang et al., 2021; Siegner et al., 2021; Standage-Beier et al., 2021; Mathis et al., 2023). Alternatively, candidate pegRNAs can be identified using large-scale screening approaches, allowing simultaneous testing of a high number of pegRNAs for new targets (Kim H. K. et al., 2021; Jang et al., 2021; Yarnall et al., 2022). However, despite the seemingly major issue of identifying new pegRNAs, several effective, therapeutically relevant pegRNAs have been reported. What is then holding up potent prime editing in HSPCs? Arguably, one important reason resides in a lack of effective vehicles for delivery of prime editors to CD34^+^ HSPCs both *ex vivo* and *in vivo*.

## Delivery is the key

A major challenge of the prime editing technology is the substantial size of the prime editor protein, which in its most optimal configuration is a 242.5 kDa protein encoded by a 6.4 kb long gene (Chen et al., 2021). Therefore, in the majority of published studies, delivery of the prime editor to human cells has been limited to transfected plasmids, either using separate plasmids encoding the prime editor, pegRNA and optionally a ngRNA, or by combining the components into single all-in-one plasmid configurations (Anzalone et al., 2019; Bosch et al., 2020; Schene et al., 2020; Kim Y. et al., 2021; Liu Y. et al., 2021; Chemello et al., 2021; Eggenschwiler et al., 2021; Kweon et al., 2021; Nelson et al., 2021; Liu N. et al., 2022; Li X. et al., 2022; Happi Mbakam et al., 2022; Hong et al., 2022; Kweon et al., 2022; Levesque et al., 2022; Peterka et al., 2022; Schene et al., 2022; Simon et al., 2022; Tao et al., 2022; Velimirovic et al., 2022; Wang et al., 2022; Zhang et al., 2022; Zhuang et al., 2022). We and others have previously utilized plasmid-based prime editors allowing stable integration of the system into the genome of human cells using piggyBac transposase, taking advantage of the prolonged expression to achieve highly effective prime editing (Eggenschwiler et al., 2021; Wolff et al., 2021). These studies also suggest that long-term expression of prime editors may be beneficial to reach editing rates that are therapeutically relevant, considering that short-term transient expression of prime editors does not always support high efficacy. However, the plasmid-based approach is a cost-effective and simple delivery platform capable of achieving high editing rates in immortalized cancer cell lines. Also, several groups have demonstrated plasmid-based delivery of prime editors to hiPSCs and hESC lines (Chemello et al., 2021; Li M. et al., 2022). Furthermore, hydrodynamic injection of plasmid DNA has also been used to deliver prime editors to the liver of mice (Liu P. et al., 2021; Jang et al., 2021). Transfection of plasmid DNA is associated with severe cellular toxicity in stem cells (Wiehe et al., 2007), and this technique is not suitable for *in vivo* use in patients.

Alternatively, a gene cassette encoding the prime editor can be delivered by exploiting the natural capacity of viruses to deliver genetic material to cells, although inherent limits on cargo size of most viral vector systems seem to limit the use of such systems for delivery of the prime editing technology. Multiple groups have, however, reported on ways of circumventing the packaging restrictions of viral vectors to deliver both the prime editor and pegRNAs. Table 1 provides an overview of the strategies utilized so far for delivery of prime editors *in vivo*. In the original work by Anzalone and colleagues, which described the first generations of prime editors, they utilized four separate lentiviral vectors to deliver an intein-split third-generation prime editor (PE3) system to mouse primary cortical neurons *in vitro* (Anzalone et al., 2019). Using a similar approach, several groups have utilized adeno-associated virus (AAV) vectors to deliver intein-split prime editing systems to cells both *in vitro* as well as *in vivo* to the mouse liver and retina (Liu P. et al., 2021; Jang et al., 2021; Liu B. et al., 2022; Böck et al., 2022; Gao et al., 2022; Grunewald et al., 2022; Zheng et al., 2022). However, editing rates have overall been modest. In an attempt to increase efficiency of dual-AAV delivery of prime editors, several studies have reported on truncated prime editors by removing the RNaseH domain of the RT with no loss in editing activity (Gao et al., 2022; Grunewald et al., 2022; Zheng et al., 2022). Using this approach, Gao and coworkers achieved up to 40% prime editing *in vitro* in HEK293T cells and 5.4% prime editing *in vivo* in mouse liver (Gao et al., 2022). However, such truncated versions still require the PE to be split into several AAV vectors, which despite optimization of intein-split sites, still comes at a cost of an overall drop of effectiveness compared to unsplit PE. An alternative approach to using intein-split prime editors was recently reported in two independent studies showing that the RT can work *in trans* with the SpCas9(H840A) nickase, allowing untethered prime editors to be delivered in two separate AAV vectors carrying SpCas9(H840A) and RT, respectively (Grunewald et al., 2022). This means that the RT in conjunction with the Cas9 nickase, but also without being physically connected to the nickase by a flexible linker, can copy the pegRNA edit sequence. This latter option opens new avenues, but also comes with the risk of overexpressing free RT and converting cellular RNAs to integration-competent pseudogenes. All in all, dual-AAV strategies have so far only shown modest editing activity *in vivo*, and they have not been used to deliver prime editors to HSPCs.

**TABLE 1:** Overview of prime editing delivery vehicles and efficacy in mouse and primary cells.

*In vitro*/*in vivo*	Delivery vehicle/method	PE system	Full length, truncated and/or split prime editor?	Cell type/tissue	Gene/Target	Efficacy	Reference
*In vitro*	Triple LV	PE3	Intein-split prime editor	Mouse primary cortical neurons	*DNMT1*	7.1%	Anzalone et al. (2019)
	IVT mRNA and synthetic pegRNAs/ngRNAs	PE3	Full-length	Patient-derived primary fibroblasts	*HEXA*	>10%	Nelson et al. (2021)
	RNPs	PE3	Full-length	Human primary T-cells	*FANCF, HEK3*	1.49%–7.54%	Petri et al. (2021)
	HDAd5/35	PE5max	Full-length	Human CD34^+^ HSPCs	*HBB*	3.4%	Li et al. (2023)
*In vivo*	Triple AAV	PE2, PE3	Split prime editor	Mouse retina	*Rpe65*	1.87%	Jang et al. (2021)
	Dual AAV	PE3	Intein-split prime editor	Mouse liver	*SERPINA1*	3.1%	Pengpeng Liu et al. (2021)
	Dual AAV	PE2, PE3	Intein-split prime editor with truncated MMLV-RT	Mouse liver	*Dnmt1, Pah* ^*enu2*^	3.4% (*Dnmt1*)>1% (*Pah* ^*enu2*^)	Böck et al. (2022)
	AdV	PE2, PE3	Unsplit truncated prime editor	Mouse liver	*Dnmt1, Pah* ^*enu2*^	35.9% (*Dnmt1*) 2% (*Pah* ^*enu2*^)	Böck et al. (2022)
	Dual AAV	PE3	Intein-split prime editor with truncated MMLV-RT	Mouse liver	*Pcsk9*	5.4%	Gao et al. (2022)
	Dual AAV	PE3	Split, untethered prime editor	Mouse liver	*Fah*	1.3%	Bin Liu et al. (2022)
	Dual AAV	PE3	Intein-split prime editor with truncated MMLV-RT	Mouse liver	*Pcsk9*	13.5%	Zheng et al. (2022)
	HDAd5/35	PE5max	Full-length	Townes mouse HSCs	*HBB*	30%	Li et al. (2023)

The table is limited to show delivery of prime editors *in vivo* in the mouse or to primary cells *in vitro*.

To circumvent the packaging restrictions posed by lentiviral and adeno-associated viral vectors, two studies have used adenovirus (AdV) to deliver prime editing drugs both *ex vivo* and an *in vivo*. Böck and colleagues initially demonstrated the use of a human adenovirus five vector to deliver an unsplit truncated prime editor *in vivo*, achieving 36% editing in mouse liver (Böck et al., 2022). Recently, a similar approach was used by Li and coworkers to deliver the most optimized prime editing system, referred to as PE5max, to human HSCs *ex vivo.* By utilizing helper-dependent adenovirus (HDAd5/35++), the authors were able to achieve 3.4% prime editing of a SCD-causing *HBB*-variant in CD34^+^ HSPCs from healthy donors and 4.6% in CD34^+^ cells derived from SCD-patients (Li et al., 2023). Importantly, the authors were able to achieve up to 40% prime editing *in vivo* in HSCs in a SCD-mouse model (CD46/Townes) by first mobilizing HSCs to the peripheral blood using G-CSF followed by a single intravenous administration of HDAd5/35++ vectors. However, the treatment strategy used *in vivo* relied on an *in vivo* selection mechanism based on resistance to O^6^-BG/BCNU (O^6^-Benzylguanine/Carmustine) in transduced cells (Wang et al., 2018).

A concern with delivering the coding sequences of genome editing tools to HSPCs is the relatively long persistence of expression of genome editing effectors, which can potentially increase the risk of off-target editing. While this has long been a concern of conventional CRISPR/Cas9, current evidence suggests that prime editing results in virtually no off-target editing, thereby potentially mitigating such concerns (Anzalone et al., 2019; Kim et al., 2020; Li M. et al., 2022; Zhuang et al., 2022). Regardless, the expression of any therapeutic genome editing drug should ideally be high, but short-lived to maximize on-target activity while minimizing off-target editing. In this regard, mRNA encoding the editing agent is potentially a powerful genome editing therapeutic. Indeed, several studies have used *in vitro*-transcribed mRNA and chemically modified synthetic pegRNAs to achieve prime editing in human cell lines (Li H. et al., 2022; Anzalone et al., 2022; Gao et al., 2022), patient-derived fibroblasts (Nelson et al., 2021), hESC lines (Li H. et al., 2022), hiPSCs (Sürün et al., 2020; Chen et al., 2021; Li H. et al., 2022), and primary human T-cells (Chen et al., 2021), often with higher efficacy than with plasmid transfection (Li H. et al., 2022). Although these efforts have focused on *in vitro* use, previous work has demonstrated that delivery of mRNA-based genome editing tools can lead to efficient editing both *ex vivo* (De Ravin et al., 2016; Newby et al., 2021; Siegner et al., 2022) and *in vivo* (Musunuru et al., 2021; Rothgangl et al., 2021). Notably, two recent studies utilized lipid-nanoparticles (LNPs) to deliver mRNA-based adenine base editors to the liver of cynomolgus monkeys, achieving notably high editing rates (Musunuru et al., 2021; Rothgangl et al., 2021). Given that delivery of mRNA-based genome editing tools to CD34^+^ HSPCs has previously been shown to be efficient and well tolerated, an mRNA-based platform of prime editing in human CD34^+^ HSPCs could very well be an intriguing approach to both *ex vivo* and *in vivo* treatment of monogenic blood disorders.

Whereas mRNA delivery still entails on-site production of the editing components, one may speculate that delivery of ready-to-use ribonucleoprotein complexes (RNPs) could potentially facilitate effective short-term editing interventions. Also, RNPs can be produced prior to treatment as a genetic drug that does not need further processing to function. For conventional CRISPR/Cas9, delivery of Cas9/sgRNA RNPs to CD34^+^ HSPCs has become the favored method for *ex vivo* genome editing, allowing high editing rates when a HDR donor template is supplied in parallel. For prime editing, *in vitro* delivery of RNPs has been demonstrated in human cells and zebrafish embryos with modest efficacy (Petri et al., 2021; Li H. et al., 2022; Liu B. et al., 2022). However, as a commercially available recombinant PE protein is not yet available, and as synthesis of the longer engineered pegRNAs (epegRNAs) is challenging and still limits availability, a wider use of this approach is currently not feasible. Furthermore, RNP-based prime editing is yet to be demonstrated *in vivo*, although we and others have previously adapted lentivirus- and retrovirus-derived particles for delivery of Cas9 protein and sgRNAs (Choi et al., 2016; Mangeot et al., 2019; Hamilton et al., 2021). Recently, such virus-like particles were successfully adapted to deliver Cas9/sgRNA RNPs to CD34^+^ HSPCs *ex vivo* (Gutierrez-Guerrero et al., 2021) as well as base editor RNPs *in vivo* to the mouse liver, achieving 63% editing (Banskota et al., 2022). Although adaptation of viral envelopes for delivery of prime editors has not yet been reported, we are likely soon to see packaging of PE-pegRNAs complexes in engineered viral particles and the use of this approach for delivery of PE RNPs.

## Outro: Will prime editing deliver in the blood?

**FIGURE 2:**
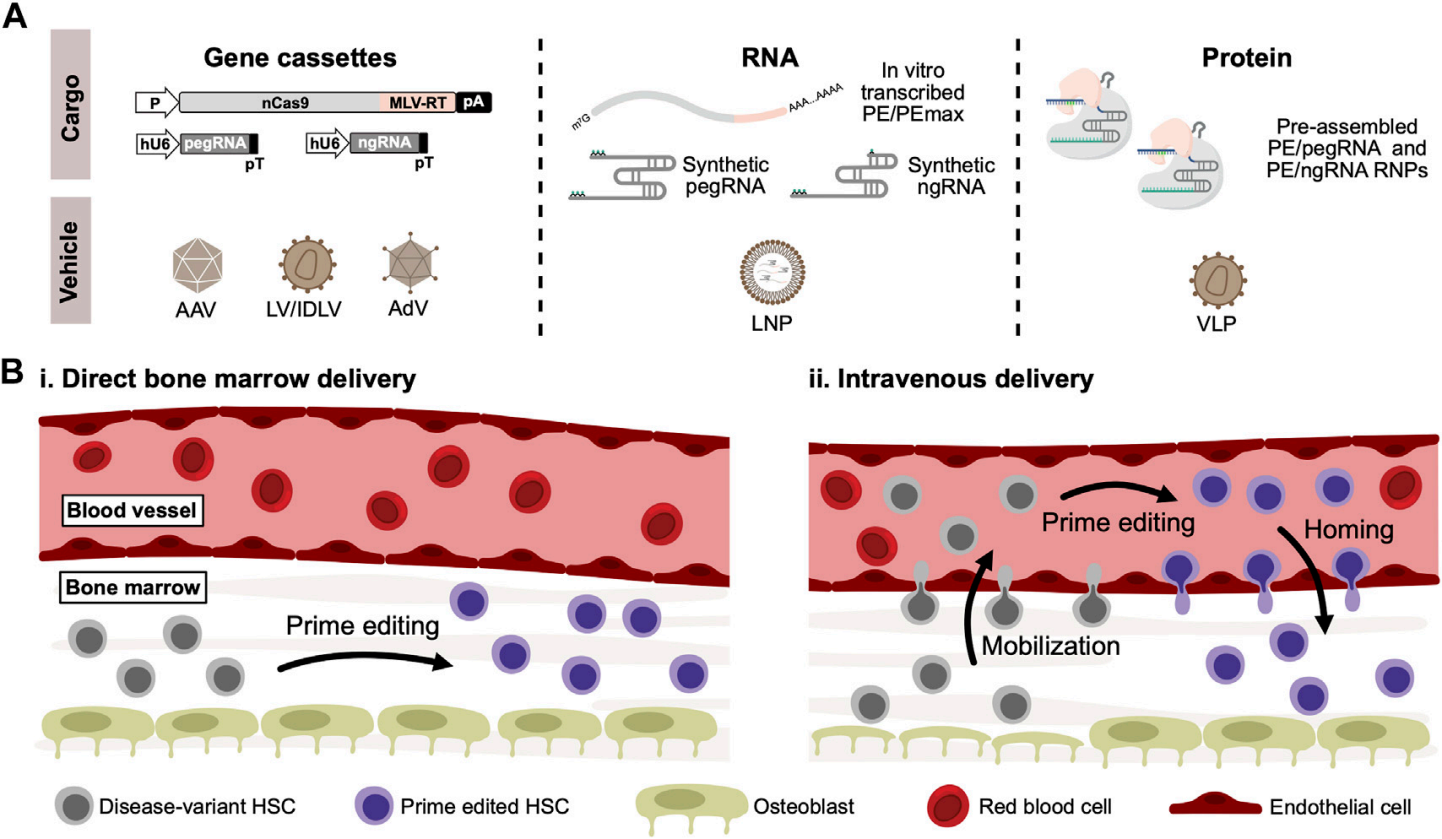
*In vivo* delivery of prime editors to HSCs. **(A).** For *in vivo* delivery of prime editors, gene cassettes coding for both the prime editor, pegRNA and optionally nicking sgRNA (ngRNA) can be delivered using viral vectors derived from adeno-associated virus (AAV) or adenovirus (AdV). Lentiviral vectors (LV) can also be used, which can alternatively be produced as integrase-deficient lentiviral vectors (IDLV) to abrogate risk of insertional mutagenesis. Alternatively, RNA-based prime editors can be delivered in lipid-nanoparticles (LNP) carrying *in vitro*-transcribed prime editors along with synthetically modified pegRNAs and ngRNAs. Lastly, delivery of ready-to-go PE/pegRNA RNPs can be accomplished through the use of virus-like particles (VLPs), typically derived from lentiviral and retroviral vector systems. **(B).** Schematic representation of potential routes for *in vivo* delivery of prime editors to HSCs residing in the bone marrow. Based on previous work on conventional *in vivo* gene therapies, two different methods can be proposed: (i) Delivery vehicles carrying prime editors are injected directly into the bone marrow, where residing disease-variant HSCs can be cured by prime editing. (ii) Alternatively, HSCs can be mobilized to the peripheral blood vessels through the use of mobilizing agents such as G-CSF or plerixafor. Delivery vehicles carrying prime editors can then be administered intravenously, allowing mobilized disease-variant HSCs to be prime edited in the peripheral blood. Prime edited HSCs can then reconstitute the bone marrow through homing.

Despite the obvious advances toward correcting disease-causing gene variants without breaking or adding DNA using prime editing, it remains relevant to ask: how can prime editors become curative drugs allowing *in vivo* genome editing therapies of blood disorders? While the currently published delivery strategies of prime editors (Figure 2A) have shown variable efficacies *in vitro* and *in vivo*, it is too early to rule any of them out. The dual-AAV delivery approach is currently the one that has received most attention for *in vivo* use due to the broad tropism and low immunogenicity (Ronzitti et al., 2020), although general low efficacy of this approach seems to be a continued challenge, possibly due to loss of activity as a result of inefficient intein-splicing (Zheng et al., 2022). However, as AAV6-serotype vectors are known to efficiently transduce human CD34^+^ HSPCs (Wang et al., 2015), further optimization of the intein-split or untethered prime editing systems could allow the dual-AAV delivery approach to be applied to CD34^+^ HSPCs not only *ex vivo* but also potentially *in vivo*. A potential pitfall of AAV-based delivery could, however, be an inadvertent ITR-mediated activation of p53-signalling in transduced HSCs, as highlighted in a recent study by Ferrari and colleagues (Ferrari et al., 2022). The AdV-based approaches potentially solve the issues of packaging restrictions that are evident for AAVs, allowing full-length prime editing systems to be packaged in a single vector. Additionally, the AdV-based approaches have so far shown the highest efficacy *in vivo,* and so far, successful *in vivo* prime editing in HSCs have only been reported using AdV. However, high immunogenicity of AdVs may raise some concerns for its safe use in the clinic (Böck et al., 2022). Delivery of mRNA- or protein-based prime editors potentially offer several advantages over viral-based DNA delivery, as the shorter duration of exposure could limit off-target effects in addition to eliminate the risk of integrating prime editor gene cassettes into the genome through recombination. For *in vivo* use, delivery of mRNA or RNPs in LNPs, virus-like particles, or other engineered formulations could possibly be an effective solution, although evidence of this is yet to be reported.

Regardless of the delivery approach, another crucial aspect of *in vivo* genome editing of blood disorders is how to make the human CD34^+^ HSPCs accessible for delivery. While intravenous administration allows efficient delivery to the liver, the HSPCs in the bone marrow are not easily accessible. Previous work using conventional gene therapies has circumvented this restriction by either directly injecting lentiviral vectors into the bone marrow (Pan et al., 2004; Worsham et al., 2006) or alternatively by mobilizing HSPCs to the peripheral blood followed by intravenous injection of viral vectors (Richter et al., 2016; Humbert et al., 2018; Li C. et al., 2021) (Figure 2B). The latter approach was used by Li and coworkers to deliver the PE5max system to HSCs *in vivo* (Li et al., 2023). For conventional gene therapies, off-target cell transduction remains a concern of these approaches. For a genome editing approach, however, these concerns might be abrogated - especially given the low by-stander and off-target editing seen with prime editors.

Although obvious obstacles remain to be overcome, one may argue that now is the time to intensify the focus on *in vivo* genome editing of HSPCs as a cure for monogenic blood disorders. The high hopes may not yet be fully supported by accomplishments in the field, and we certainly need more evidence of efficacy and safety. Nevertheless, recent advances in site-targeted gene correction for blood disorders combined with engineering of new technologies, like prime editing, that are constantly refined and upgraded, seem to breed optimism and willingness to invest. The interest is certainly there, as illustrated by Prime Medicine, a company that specifically seeks to develop prime editing-based treatments of diseases, becoming one of the largest biotech IPOs of 2022. Challenges remain, but prime editing may represent a safe and efficient way of treating diseases of the blood directly in the patients. We are not quite there yet, but further efforts towards developing efficient *in vivo* delivery platforms could make prime editing the closest we have yet been to a true drug that can be administered to humans to correct disease-causing gene variants without supplying additional DNA.
